# Vitamin D and physical activity as co-modifiers of muscle health and function - a narrative exploration

**DOI:** 10.1097/MS9.0000000000003502

**Published:** 2025-06-25

**Authors:** Inshal Jawed, Hafiza Quratul Ain, Farah Abdul Razaq, Muhammad Umair Qadir, Shafaq Jabeen, Farah Alam, Maham Javaid, Mehak Mobin, Danaish Kumar, Ruhul Rai, Syed Ali Farhan Abbas, Abu Huraira Bin Gulzar, Agha Muhammad Wali Mirza, Salma S. Alrawa, Wajeeha Imam

**Affiliations:** a Department of Medicine Dow University of Health Sciences, Karachi, Sindh, Pakistan; b Department of Medicine Combined Military Hospital, Multan, Punjab, Pakistan; c Department of Medicine Civil Hospital, Karachi, Sindh, Pakistan; d Department of Medicine Karachi Medical and Dental College, Karachi, Pakistan; e Department of Medicine Manor Hospital, Walsall, UK; f Department of Medicine Allama Iqbal Medical College, Lahore, Pakistan; g Department of Medicine Medicare Hospital, Sohail University, Karachi, Pakistan; h Department of Medicine Liaquat University of Medical and Health Sciences, Jamshoro, Pakistan; i Department of Medicine Jinnah Medical & Dental College, Karachi, Sindh, Pakistan; j Department of Medicine Services Institute of Medical Sciences Lahore, Pakistan; k Faculty of Medicine, University of Khartoum, Khartoum, Sudan

**Keywords:** bone, muscle strength, physical activity, vitamin D

## Abstract

Bone health requires different factors to grow. One of the main requirements is vitamin D. Much of the regulation of calcium levels also affects muscle strength and function, protein synthesis, and other cellular activities. Furthermore, the benefits of vitamin D on the muscular system are comparable to those of physical activity in terms of shaping the structural and functional changes in muscles. Our paper focuses on the synergistic effects of physical exercise and vitamin D on metabolism how they can impact muscle health, including the physiological rationale, outcomes of its deficiency, and measures for counteraction thereto. Here, we outline the effect of combined interventions for optimizing muscle strength.

**HIGHLIGHTS**

- This narrative review highlights the synergistic relationship between vitamin D and physical activity in promoting muscle health, with a particular focus on the physiological mechanisms, the implications of deficiencies, and practical recommendations for optimization.
- Muscle health is a cornerstone of physical performance and overall well-being. This article synthesizes existing clinical research to elucidate how vitamin D metabolism and physical activity interact as co-modifiers, shaping muscle strength and function.
- The narrative approach provides a comprehensive yet accessible synthesis of relevant literature, contributing valuable perspectives to ongoing discussions in the field.

## Introduction

Vitamin D is proven to keep humans metabolically healthy, enables them to undertake basic activities independently, and reduces the incidences of falls and related injuries for those aging. Furthermore, it helps in physical and overall well-being. Among the myriad factors influencing muscle health, vitamin D and physical activity stand out as indispensable contributors. On the other hand, exercise, especially strength training, has been acknowledged historically as a fundamental basis for the development and maintenance of muscle and motor units. However, the combined effect of these two factors indicates that there is an even bigger potential for improving the condition of muscles. Vitamin D deficiency is widespread throughout the world and is frequently linked to weariness, sarcopenia, and muscular weakness. Dietary deficiencies, modern lifestyles, and little solar exposure make this insufficiency worse. Regular exercise, on the other hand, has been demonstrated to lessen these negative consequences by reducing the chance of chronic illnesses, including diabetes, cardiovascular disease, and osteoporosis, in addition to increasing muscle strength. In addition to reviewing current clinical evidence on both the physiological processes involved in muscle metabolism and the anti-athletic effects of vitamin D, this review aims to explore possible synergies of vitamin D and exercise to maximize muscle and bone outcomes.

## Detailed metabolism of vitamin D

Vitamin D is metabolized through an intricate, multi-step pathway vital for preserving musculoskeletal integrity, calcium homeostasis, and many other physiological processes. First, it comes from the skin converting vitamin D3 (cholecalciferol) to skin exposed to ultraviolet B (UVB) light or ingesting vitamin D2 (ergocalciferol) or D3 in food. These precursors are inert biologically but like molecules that undergo successive hydroxylations to become metabolically active. Initial hydroxylation by the liver first gives vitamin D its form as calcidiol, or the 25 hydroxyvitamin D [25(OH)D]. The form mentioned above is the main circulating reservoir of vitamin D and is most often considered a marker of vitamin D status^[1,2]^.

**Figure 1.**
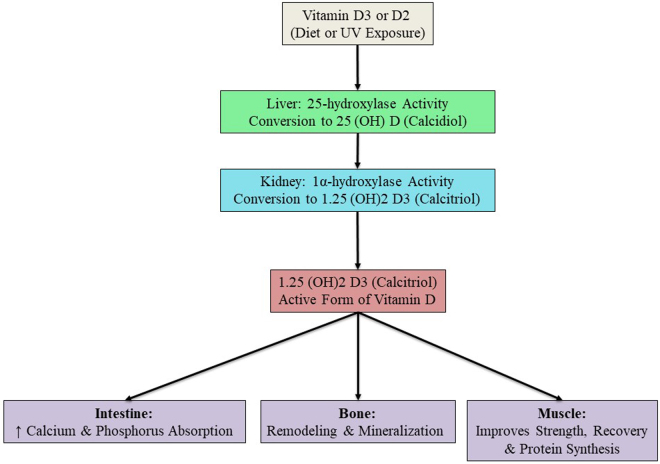
Metabolism of vitamin D—flowchart.

The second hydroxylation is typically catalyzed by 1α hydroxylase (CYP27B1) in the proximal tubules of the kidneys and is the site of production of the physiologically active metabolite 1,25 dihydroxy vitamin D (calcitriol)^[1,2]^. Calcitriol, when it binds to the vitamin D receptor (VDR), helps us understand how it works. It is expressed in numerous organs, such as kidneys, intestines, bones, and skeletal muscle^[3]^. This receptor‐ligand interaction facilitates the transcription of target genes related to muscle function, bone remodeling, and calcium and phosphate absorption^[3]^ (Fig. 1).

In addition, there is new evidence that dihydroxy vitamin D is synthesized not only in kidneys but also in other tissues such as macrophages and the placenta and that calcitriol can exert strong paracrine action in particular pathological conditions^[3,4]^. The metabolism of vitamin D is also controlled by feedback inhibition. Increased calcitriol levels increase the expression of 24 hydroxylases (CYP24A1), an enzyme involved in 1,25 dihydroxy vitamin D metabolism, to inactivate metabolites and downregulation of 1 alpha-hydroxylase activity^[1]^.

Calcitriol both regulates calcium influx and enhances mitochondrial function, promoting protein synthesis via activation of pathways such as Akt/mTOR signaling^[3]^ in muscle cells. Vitamin D intake is always important for muscle contraction, repair, and growth. Furthermore, recent studies have demonstrated that variation within the VDR gene may occur prior to variation in vitamin D sensitivity and may be associated with muscle strength and other physical performance measures^[3,5]^.

**Figure 2.**
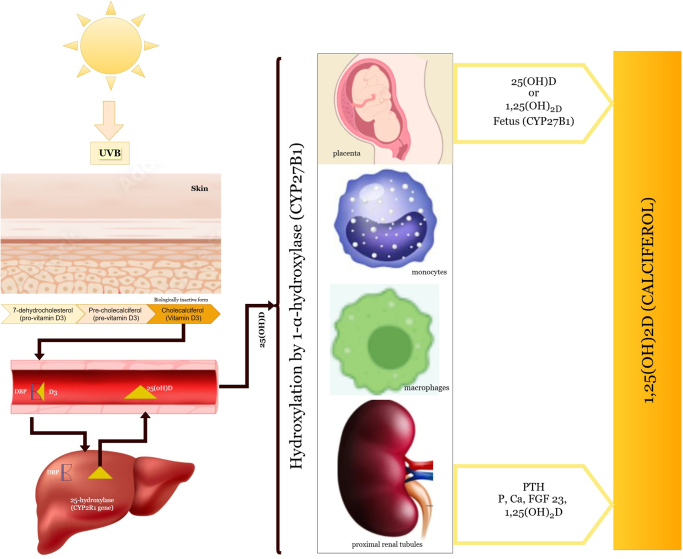
Metabolism of vitamin D—graphical presentation.

Suitable vitamin D intake is necessary in aiding muscle contractions, repair, and growth. Moreover, recent research suggests that genetic variation in the VDR gene may actually precede variation in vitamin D sensitivity, with implications for muscle strength and physical performance measures^[3,5]^ (Fig. 2). A recent study by Qi *et al*^[6]^ concluded that oral supplementation of vitamin D along with health education and lifestyle modification can have significant impact on middle-aged and elderly individuals. Their retrospective, propensity score-matched study observed clear differences in the intervention group; improved lower limb strength by +20N (*P*-value ≤ 0.05), improved grip strength by 2.4 kg (*P*-value <0.05), and improved pinch strength by 0.6 kg (*P*-value <0.05)^[6]^. Another systematic review and meta-analysis by Xiong *et al*^[7]^ suggested the significant benefits of vitamin D analogs in elderly people (aged >58 years). They reported a significant increase in quadriceps muscle strength after supplementation thus reducing the risk of falls by 19% (RR: 0.81, 95% CI: 0.67–0.98)^[7]^. Thus, current evidence suggests a strong association of vitamin D supplementation with the strength of muscle in middle-aged to elderly people.

## Parathyroid hormone’s function in the metabolism of vitamin D

Vitamin D metabolism also requires parathyroid hormone (PTH), in addition to maintaining calcium and phosphate balance. A release of PTH is secondary to the parathyroid glands when serum calcium decreases below the physiological threshold.

This hormone is stimulatory for 1α-hydroxylase (CYP27B1) and is primarily operative in the kidney. It takes the main form of vitamin D circulating in the body, 25 hydroxyvitamin D [25(OH)D], and changes it to its active form, 1,25 dihydroxyvitamin D (calcitriol)^[5]^. This, in turn, stimulates the production of the calcium-binding protein calbindins D, and at higher calcitriol levels, intestinal calcium absorption is upregulated^[8–10]^.

In addition to promoting intestinal calcium absorption, calcitriol influences bone resorption by acting on osteoclasts. It stimulates by activating the bone-resorbing cells, while inhibiting osteoblastic calcium transfer from the bone matrix to cells, thus, restoring calcium homeostasis by releasing calcium and phosphate into the bloodstream^[8]^.

The finding of a dual action for PTH, increasing plasma calcium concentrations alone by analogy to vitamin D, underscores a cooperative interaction between PTH and vitamin D in maintaining systemic calcium levels.

One indirect mechanism of PTH regulation is for 25(OH)D availability for activation. PTH decreases renal excretion of phosphate and increases the renal reabsorption of filtered calcium, thereby assuring the necessary substrate availability for optimal calcitriol synthesis^[8]^. Furthermore, feedback mechanisms carefully regulate this process because high calcitriol levels prevent hypercalcemia and preserve a delicate physiological balance by suppressing additional PTH secretion through negative feedback loops.

Calcium modulation is beyond PTH’s scope: new research tells us more about PTH’s role in the metabolism of vitamin D.

For example, chronic elevation of PTH, as seen in secondary hyperparathyroidism associated with chronic kidney disease (CKD), has been shown to disrupt normal feedback regulation of 1α-hydroxylase and, therefore, impaired calcitriol production and associated musculoskeletal complications^[3,8]^. A study by Dong *et al*^[11]^ demonstrated that ubiquitin-proteasome pathway contributes toward muscle wasting due to raised PTH levels in CKD. They found that increased PTH leads to the expression of E3 ubiquitin ligases that cause enhanced protein degradation leading to muscle wasting^[11]^. This underscores the need to control pathological conditions associated with both PTH dysregulation and pathological vitamin D status as is reported by Wang *et al*^[12]^. They observed that vitamin D supplementation is significantly associated with a 22% reduced relative risk of muscle deterioration in end-stage renal disease patients (RR: 0.78, 95% CI: 0.16–0.68, *P*-value = 0.003).

As a result, PTH plays a central role in the tightly connected system of vitamin D bioavailability, which is different from calcium-phosphate homeostasis and involves other processes in bone remodeling and musculoskeletal dysfunction. These regulatory routes detail the integrated responses between hormonal cues and vitamin D in preserving the integrity of the musculoskeletal system.

## Metabolism of vitamin D in renal failure

Vitamin D metabolism is disturbed in CKD by decreased 1α-hydroxylase activity. This leads to secondary hyperparathyroidism, calcemic imbalance, and musculoskeletal consequences such as osteomalacia and muscle weakness^[9]^. Consequently, many CKD patients require active vitamin D analog or calcitriol supplementation to combat this effect.

## Functions of vitamin D

Vitamin D has other very important roles beyond calcium and phosphorus regulation:

- Calcium and phosphorus regulation: They are involved in intestinal absorption of calcium and phosphorus by affecting the intestinal synthesis of these substances (sodium phosphate cotransporters and calcium-binding proteins) in response to vitamin D. All of this helps keep bone mineral density and muscle contractility in optimal condition^[2]^.
- Immune system modulation: Spontaneous monocyte differentiation into macrophages, interaction with T cells, and regulation of T cell activity support monocytes’ capacity to enhance both innate and adaptive immune responses. These components have also been proven to support the body’s defenses against autoimmune conditions such as multiple sclerosis and rheumatoid arthritis^[13]^.
- Muscle function: Vitamin D is needed for muscle recovery and strength. It enhances calcium influx through muscle cells and, thus, improves excitation-contraction coupling. Also, it plays a role in muscle development and repair by activating signaling pathways that raise protein synthesis (e.g. Akt/mTOR)^[3]^.

Inflammation control: Vitamin D lowers chronic low-grade inflammation by upregulating anti-inflammatory markers and suppressing proinflammatory cytokines such as TNF-α and IL-6. This is essential for tissue repair and recovery following injury or exercise^[14]^.

Vitamin D modulates apoptosis and cell cycle progression to reduce cancer risk. In particular, it causes growth inhibition in the cells of the tissues of the breast, prostate, and colon and promotes their differentiation^[4]^.

- Cardiovascular health: Recent studies have shown that vitamin D regulates blood pressure, vascular smooth muscle contraction, and endothelial function, all of which contribute to cardiovascular health.

Its insufficiency has been associated with hypertension and an elevated risk of atherosclerosis^[4,9]^.

That alone shows just how important vitamin D is for general health. Nevertheless, not having enough vitamin D in our diet, from supplements or (rarely) from the sun, can have negative effects on the skeleton and muscles and increase the risk of certain chronic diseases.

## Biochemical mechanisms

- Phosphorus and calcium metabolism: Vitamin D is well known to impact the absorption of calcium, and hence bone health and the contraction of muscles. Vitamin D keeps calcium and phosphorus levels optimal, so the musculoskeletal system remains structurally and functionally intact^[2]^.
- Inflammatory modulation: Chronic inflammation, often exacerbated by physical inactivity, is mitigated by vitamin D’s immunomodulatory effects. Inhibition of the expression of proinflammatory cytokines, such as tumor necrosis factor-α (TNF-α) and interleukin-6 (IL-6), is observed; vitamin D fosters a conducive environment for muscle repair and regeneration^[13]^.
- Mitochondrial function: Studies over the last few years have shown that vitamin D may promote mitochondrial biogenesis and function, an important step in sustained energy production required during prolonged physical activity^[15]^. This effect is most profound in populations with mitochondrial dysfunction or metabolic disease.

## Vitamin D insufficiency conditions

Vitamin D deficiency has been connected to the pathophysiology of numerous medical conditions because of its systemic significance:

- Musculoskeletal disorders: A lack of vitamin D keeps calcium and phosphate from functioning properly in the body – so the bone does not mineralize normally, leading to osteomalacia or rickets.

Long-term deficiency aggravates age-related bone loss and sarcopenia, thereby predisposing the elderly to an increased risk of fracture and functional decline^[2,3]^.

- Metabolic disorders: Vitamin D insufficiency has been linked to higher rates of type 2 diabetes, insulin resistance, and obesity, and it is related to the pathophysiology of metabolic syndrome as well as its role in systemic inflammation and adipocyte differentiation^[16]^.
- Immune dysregulation: Vitamin D deficiency decreases macrophage activity and a T cell response, therefore weakening the immune system. Along with this, people become more susceptible to infections and autoimmune conditions like rheumatoid arthritis, multiple sclerosis, and inflammatory bowel disease^[13]^.

- Cardiovascular diseases: It guarantees the preservation of ideal muscle contractility and bone mineral density^[2]^. Low vitamin D levels have also been shown to lead to arterial stiffness, endothelial dysfunction, and hypertension. These factors lead to higher cardiovascular morbidity and death as well as atherosclerosis^[17]^.
- Neurological disorders: Vitamin D influences brain health through neuroprotective mechanisms, including the regulation of neurotrophic factors and calcium signaling. Deficiency has been associated with cognitive decline, dementia, and mood disorders (e.g. depression)^[18]^.
- Infectious diseases: Vitamin D also belongs to innate immunity, which is strengthened by the amplification of antimicrobial vaccines such as cathelicidin. Deficiency has been associated with severe respiratory infections, including a heightened risk of complications from COVID-19^[19]^.

The broad ramifications highlight the necessity of maintaining sufficient vitamin D levels for a number of physiological system health benefits. To counter its far-reaching health effects, you must address deficiency through supplementation, changes in diet, and improvements in lifestyle.

## Physical activity and muscle strength

Muscle strength and function are important components of overall health. Getting your vitamin D levels optimally meets your needs, and it works synergistically with physical activity to promote optimal muscle performance and recovery. Here, we explore the types of physical activities that enhance muscle health, the mechanisms of how physical exercise and vitamin D work together to make you stronger, and how they can work together to help you achieve your goals.

### Different forms of exercise

Different forms of physical activity target various aspects of muscle health and overall functionality:

- Resistance training: Building muscles and strength begins with weight lifting and resistance bands. This stress produces mechanical stress, which induces the mechanism of repair and, hence, muscle hypertrophy^[20]^.
- Aerobic exercise: Running, cycling, swimming, and other activities increase cardiovascular health and endurance and improve oxygen and nutrient delivery, all of which support muscle function^[5]^.
- Functional training: Exercising activities that mimic your daily movements, such as squatting, lunges, and step-ups, improves your balance and coordination and lowers your chances of falling, especially in older adults^[21]^.
- High-intensity interval training (HIIT): HIIT is a quick and effective way to improve cardiovascular and muscular responses. It involves intervals of high-intensity exercise and rest^[5,22]^.
- Flexibility and stretching exercises: Practices such as yoga and Pilates make the muscles more elastic and joints more mobile, decreasing the chances of injury^[8]^.
- Weight-bearing activities: Walking, jogging, or dancing offers a total musculoskeletal benefit because they work both the muscles and the bones^[21]^.

## Mechanisms enhancing muscle strength

Complex biochemical and biomechanical processes mediate the physiological adaptations resulting from exercise:

- Pathway activation: Among the pathways that are activated when you exercise, for example, is the signaling mechanism known as insulin-like growth factor 1 (IGF-1)^[14]^.
- Mechanical tension-induced remodeling: Mechanical tension, which occurs from resistance exercises, promotes remodeling and hypertrophy of a muscle fiber^[20]^.
- Improved blood flow: It is also great for bringing an adequate supply of oxygen and nutrients, oxygen being vital for the growth and repair of muscle, while the required nutrients like iron are essential for maximum operation of muscles^[5]^.
- Vitamin D interplay: Vitamin D is important in the optimal functioning of these mechanisms to maintain calcium homeostasis, enhance muscle contraction, and reduce inflammation^[1,2]^.

## Synergistic effects of physical activity and vitamin D

The interaction between vitamin D and physical activity represents a multifaceted relationship that is pivotal for optimizing musculoskeletal health and overall physiological well-being. Research shows you also need vitamin D for best muscle function and recovery, and evidence suggests that it does so in sympathetic partnership with exercise.

- Flexibility and balance: For older adults who may be prone to falling, yoga stretching exercises, in addition to realizing enough vitamin D, will keep muscles from tightening and will guard against imbalance.

- Weight-bearing activities: Vitamin D supplements the recovery of bones and muscles after activities such as jogging and dancing that stress them^[19]^.

- Functional movements: Vitamin D and daily task-oriented exercise can enhance neuromuscular coordination and reduce falls and fractures^[23]^.

- Faster recovery: Vitamin D’s anti-inflammatory qualities are important for decreasing exercise-induced muscular damage. Vitamin D attenuates inflammatory cytokines and oxidative stress markers and hence helps with post-exercise muscle repair, reducing the recovery time^[9]^. Furthermore, its activation of the modulation of the nuclear factor-κB (NF-κB) pathways confirms its reparatory capacity^[8]^.

- Improved training response: Optimized vitamin D levels are associated with increased exercise-induced muscle hypertrophy and strength gains. It has been shown to mediate its effect through calcium homeostasis, a function necessary for muscle contraction^[1]^, and activation of anabolic pathways. Most importantly, this vitamin can increase muscle mass and strength by stimulating IGF-1, which prompts protein synthesis^[17]^.

- Enhanced regeneration: Vitamin D sharply and profoundly enhances the activation of satellite cells, precursor cells (typically quiescent) critical for muscle repair and regeneration. Vitamin D improves satellite cell proliferation and differentiation, hastening recovery through genomic and nongenetic mechanisms^[3,24]^.

**Figure 3.**
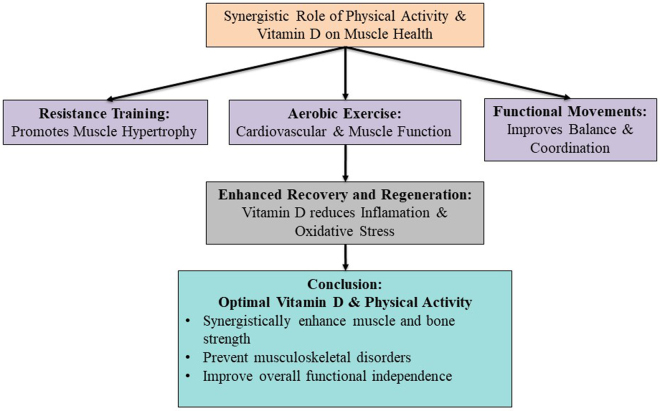
Synergistic role of vitamin D and physical health on muscle health.

The overall idea is to optimize muscular health through adequate physical exercise and adequate blood vitamin D levels. Demonstrating the beauty of this synergy has successfully earned increases in strength, better coordination, and decreased risk of injury^[21]^ (Fig. 3).

## Clinical evidence

Many studies have been done on the type and quantity of vitamin D supplements to take when regular exercise is being done in populations at risk of muscle weakness (sarcopenia).

- Impact on older adults: Supplementary vitamin D is beneficial for physically active older adults in maintaining muscle strength. Given vitamin D’s ability to increase neuromuscular function and decrease the rate of muscle protein degradation^[8,10]^, this effect is attributed. Additionally, these benefits are further increased with resistance or weight-bearing exercises^[21]^.

- Reduced fall risk: At the comprehensive meta-analysis level, integrating vitamin D supplementation into structured exercise programs lowers fall risk in older adults. The overlapping effects among the participants suggest that this may result from improved balance, postural control, and increased muscle function^[19]^.

- Elite athletes and physical performance: The benefits of vitamin D for athletes are well-documented. Studies show that increased vitamin D levels can greatly increase key performance metrics, such as sprint speed and maximum oxygen uptake^[24]^.

- Chronic conditions and recovery: Furthermore, Vitamin D has a function in persons dealing with chronic circumstances or surgeries. For instance, in stroke survivors, supplements of low-dose vitamin D reduced fall rates and diminished muscle atrophy, highlighting its rehabilitative potential^[23]^.

## Practical implications

- Supplementation guidelines: To be effective, vitamin D supplements require raising and maintaining serum 25 hydroxyvitamin D levels above 30 ng/mL without adverse effects, and doses of 800–2000 IU daily improve musculoskeletal health^[9]^.

- Integration with exercise: Exercise regimens that utilize structured exercise that includes resistance and weight-bearing activities bolster the benefits of vitamin D supplement use. Aerobic training programs for cardiovascular health or resistance training to stimulate muscle hypertrophy are individualized and thus interact synergistically^[16]^.

- Population-specific strategies: Targeted intervention is needed for optimal vitamin D levels among vulnerable populations, including older adults who spend longer indoors or get less sunlight due to illness or other medical conditions and people with chronic diseases^[25]^.

## Combined interventions for optimized musculoskeletal health

### Dietary adjustments

Dietary changes are the base for increasing vitamin D. Good sources of vitamin D are egg yolks, fortified dairy products, and fatty fish (such as salmon, mackerel, and sardines), which are recommended for their bioavailability and role in maintaining serum vitamin D levels^[2,9]^. Finally, if one does not get enough sun or dietary vitamin D, start taking supplements^[25]^. Research via meta-analyses shows that regular supplementation is an effective means to increase musculoskeletal strength and general metabolic health^[8,14]^.

### Exercise protocols

Maintaining and improving musculoskeletal health requires exercise interventions. Combined protocols mixing resistance training, aerobic activities, and functional exercises have been shown to produce breakthroughs in increased muscle mass, strength, and global physical performance^[5]^. In the case of younger, physically active people, additional benefits for improving insulin sensitivity and metabolic outcomes have been shown in HIIT^[5]^.

### Sunlight exposure

However, encouraging adequate sunlight exposure remains a natural and cost-effective strategy for increasing naturally produced vitamin D. Peak sunlight hours are the time of day to get outdoors for a vitamin D boost^[2,25]^. While this approach may be ideal, it should be balanced out with skin cancer risk, and exposures should be short, frequent, and not prolonged, like sunbathing^[9]^.

### Regular monitoring

Periodic assessments of serum 25-hydroxyvitamin D concentrations ensure tailored interventions. Regular monitoring allows clinicians to adjust supplementation and dietary plans based on individual needs, preventing both deficiency and toxicity^[24]^.

## Special populations

### Older adults

Resistance training and vitamin D supplementation together have demonstrated strong benefits in lowering fall risk and improving sarcopenia in older persons^[8,19]^. Age-related muscle weakness and waste, commonly referred to as sarcopenia, can substantially influence quality of life. Vitamin D preserves muscle function and improves gait and balance, consequently reducing fall-related injuries, as documented by systematic reviews^[21,26]^.

### Athletes

Age-associated muscle wasting, weakness, or sarcopenia can have a harsh impact on quality of life. However, systematic reviews have shown that vitamin D preserves muscle function and improves gait and balance, consequently reducing fall-related injuries^[21,26]^.

### Individuals with chronic conditions

Diet and exercise can correct vitamin D insufficiency in diabetes, HIV, and cardiovascular disease, improving fat mass and metabolic parameters^[9,16]^. In these populations, the relationship of exercise to augmented physical function decreased systemic inflammation, and improved glycemic control is also present^[16]^.

## Emerging evidence and future directions

New research shows that vitamin D is not just for bones. Vitamin D deficiency has been linked to autoimmune disorders, so it is necessary to manage it proactively^[13]^.

Furthermore, recent research indicates that vitamin D supplementation, together with physical activity, could act synergistically, resulting in neuromuscular benefits, decreased fracture rates, and an increase in quality of life^[14,19,23]^.

The best practice for wide-ranging populations focuses on comprehensive strategies, including dietary modification, highly structured exercise programs, consistent sunlight exposure, and regular monitoring of musculoskeletal health. Moreover, to improve the outcomes, future research should further examine minority reporting within an intervention model, which also accounts for an individual patient’s genetic predispositions, lifestyle factors, and comorbidities – all hallmarks of precision treatment.

## Summary of current evidence on vitamin D and muscle strength

Despite the beneficial effects of the vitamin D, the literature is uncertain about its satisfactory clinical advantages. Various meta-analytic studies pooled the results of the current literature trying to find out a conclusive evidence but the results are inconclusive. A recent randomized control trial (RCT) by Houston *et al*^[27]^ concluded that supplementation of vitamin D showed inconclusive results; low-functioning older adults did not benefit from 2000 IU/d vitamin D3. No significant improvement was seen in leg power, strength, or physical performance. Similarly, a non-randomized controlled trial, using the quasi-randomization method reported insignificant effects of vitamin D supplementation in healthy older adults who had no prior history of being involved in sports or resistance training over the past year. They observed no enhancement in gain of muscle strength, lean body mass or decrease in fat mass. Furthermore, there was no improvement in cardiorespiratory fitness^[28]^. However, interestingly a meta-analytical study done on RCTs by Samantha Harrison *et al*^[29]^ included 6481 older patients aged ≥60 years. Their results show that vitamin D supplementation had a positive effect on the muscle mass (SMD(Standardized Mean Difference): 0.27, 95% CI: 0.1–0.42, *P*-value <0.001). They highlighted that vitamin D supplementation can be a potential solution for the management of sarcopenia, however, future studies shall focus on dosing strategies and treatment duration^[29]^. These results are backed by several other studies like Qi *et al*^[6]^, Wang *et al*^[12]^, Dong *et al*^[11]^. However, an important point to consider is that, all of these studies were done on different population groups. Either they involved the normal population showing insignificant results entirely or else on chronically ill patients showing significant improvement. Thus, future research shall focus on specified populations who can be benefited from this combined therapy.

## Future directions for research in vitamin D and muscle health

It is becoming clear that approaches to vitamin D supplementation should be individualized, as people will have wildly different responses depending on prior vitamin D levels, genetics, and physical activity^[15]^. Further studies should assess proposed supplementation doses adapted to various levels of activity and demography, including regions where sunlight exposure is lacking^[25]^. The researchers use data from controlled trials^[26,30]^ and systematic reviews^[14]^ to refine the guidelines on how to maximize muscle strength and physical performance effects.

### Genetic factors

On the flip side, vitamin D responsiveness is itself a burgeoning area of research, and this is because we still do not understand the genetic underpinnings of how your body responds to vitamin D. Genetic variations in VDR genes have been shown to influence individual’s response from supplementation^[3]^. Genetic influences could be unraveled through advanced genomic techniques, such as genome-wide association studies^[1]^, which aid clinicians in predicting responsiveness to vitamin D interventions so that more effective, patient-specific treatment strategies can be developed.

### Biomarkers development

Importantly, biomarkers must be found to assess how vitamin D influences muscle health under physiological conditions. Biomarkers currently measured (serum 25 hydroxyvitamin D) are useful but not sensitive and specific for muscle outcomes^[14]^. Future research should focus on the development of novel biomarkers directly informing the measurement of muscle metabolism, strength, and recovery during the demand and recovery processes^[24]^.

### Technological integration

In this field, the integration of Wearable technology and artificial intelligence (AI) is a breakthrough. Wearable devices can monitor physical activity, sun exposure, and medication compliance, and AI can interpret this data to tailor vitamin D supplementation and exercise plans made just for each individual^[23]^. This would probably heighten efficacy and adherence, too, especially in at-risk groups such as the elderly and people with long-term illnesses^[21]^.

### Policy advocacy

Vitamin D insufficiency is a public health concern since vitamin D insufficiency is associated with musculoskeletal diseases, falls, and fractures^[19]^. They need to be effective advocates for the proper policies that ensure awareness and supplement programs. Wide adoption of supplementation guidelines can be driven by collaborative efforts between governments, healthcare providers, and public health organizations, especially in high-risk populations with restricted sun exposure^[9]^.

### Novel exercise regimens

Vitamin D insufficiency is a public health concern since it is associated with musculoskeletal diseases, falls, and fractures. While traditional resistance training has been well-studied, future research should explore hybrid training models that combine resistance, flexibility, and balance exercises to optimize muscle strength and overall function^[21]^. For example, the regimens may be especially beneficial for older adults and those recovering from musculoskeletal injuries^[18]^.

### Addressing special populations

Depending on the population (e.g. athletes and people with chronic illnesses), individual approaches may be necessary in some circumstances to meet the unique requirements of this demographic. These results are in line with both of these, suggesting that higher dosages of vitamin D supplementation may boost performance and recovery in athletes^[24]^. Just as targeted interventions to build muscle mass and reduce the risk of falls in people with HIV or recovering from strokes are possible^[16,23]^. However, these findings require further investigation through randomized controlled trials and longitudinal studies.

## Conclusion

The synergistic relationship between vitamin D and physical activity plays a pivotal role in optimizing muscle health, with profound implications across diverse populations and life stages. It controls the calcium homeostasis, aids in protein synthesis, modulates immune response, and supports recovery and regeneration of skeletal muscle (as reviewed here). When combined with targeted physical activity – ranging from resistance training to weight-bearing and functional exercises – vitamin D supplementation has a potential effect on muscle strength, coordination, and overall functionality. Combined strategies for these key factors are particularly important among vulnerable populations (such as older adults, persons with chronic diseases, and those with limited outdoor exposure) to decrease the risks and complications.

Future directions include new biomarkers for vitamin D, customized exercise supplementation regimens, and eventually an interdepartmental approach toward combined intervention for musculoskeletal health. Moreover, further studies are needed with robust designs targeting older population with chronic diseases along with special emphasis on its dosage and treatment strategy.

## Data Availability

Not applicable.
